# Supplementary material for: Are carbon emissions trading and green financial instruments synergistic? -Comprehensive quantitative research based on content analysis
Source: PLoS One. 2024 Mar 7;19(3):e0298601. doi: 10.1371/journal.pone.0298601 (PMC10919601; doi:10.1371/journal.pone.0298601)
Supplement: S1 Data — (ZIP) [file pone.0298601.s001.zip › Data/地级市Z_F工作报告.zip!/░▓╗╒/┴∙░▓╩╨/└┤╫╘╣1⁄2╓┌║┼í╛┬φ┐╦╩2╛▌═°í┐_2006.docx]

各位代表：
　　现在，我代表市人民政府，向大会作政府工作报告，请予审议，并请市政协委员和其他列席人员提出意见。
　　一、全市人民团结拼搏，取得了“十五”及2005年工作的显著成就
　　“十五”是建市后的第一个五年。五年来，在省委、省政府和市委的坚强领导下，全市人民高举邓小平理论和“三个代表”重要思想伟大旗帜，坚持发展第一要务，坚持招商引资主战略和工业化核心战略，坚持基础设施建设先行和城乡统筹，以适度超前的城镇化，带动相对滞后的工业化，促进农业产业化，加速信息化，团结奋斗，攻坚克难，实现了“前三年打基础，后两年快发展”的预期目标，胜利完成了“十五”计划。综合实力显著增强。生产总值由2000年的170.9亿元增加到2005年的312.8亿元，年均增长9%，人均达到611美元；财政收入同口径年均增长 9.2 %。结构调整进展明显。三次产业比重由37.5∶31.5∶31调整为26.7：33.5：39.8；规模工业增加值年均增长14.3%，对经济增长贡献率达到24.3%。固定资产投资不断加大。累计完成378亿元，是“九五”的1.6倍，宁西铁路建成通车，临淮岗工程基本完工，一批事关发展的重大项目相继竣工投产，交通、水利、通信状况全面改善。城乡面貌发生深刻变化。中心城市建成区面积和人口规模实现翻番，城镇设施逐步改善，城镇化率由18%提高到28.7%。对外开放成效明显。招商引资累计到位境内资金144亿元、利用境外资金1.7亿美元；累计出口5.4亿美元，是“九五”的2.5倍。城乡居民生活水平有较大提高。农民人均纯收入由1571元增加到2255元，城镇居民人均可支配收入由4304元增加到7810元，分别年均增长7.5%、12.7%；城乡居民储蓄存款余额由81.5亿元增加到208.1亿元，增长1.6倍。总体来说，这五年是我市经济总量较快增长、综合实力明显增强的五年；是改革开放步伐加快、发展基础不断夯实的五年；是城乡面貌发生很大变化、人民群众得到更多实惠的五年。
　　2005年是“十五”的最后一年，也是新一届的开局之年。全市人民认真贯彻市第二次党代会精神，团结奋斗，开拓进取，圆满完成了市二届人大一次会议确定的目标任务。
　　经济发展步伐加快，人民生活水平不断提高。生产总值增长12.1%。财政收入18.7亿元，增长16.1%，同口径增长26.6%。农业总产值增长5.7%；产业化经营步伐加快，12个优势产业带基本形成，国家和省级龙头企业增加到14户，首批确定了30户市级龙头企业，“三农”公司等龙头企业应运而生，霍邱县被授予全国粮食生产先进县标兵。规模工业增加值增长28.3%；效益综合指数129.3，比上年提高7.7个百分点。社会消费品零售总额119.4亿元，增长12.4%。年末金融存款余额293.4亿元，比年初增加48.1亿元。全年接待游客263万人次，实现旅游总收入12亿元。城镇居民人均可支配收入同口径增长13.2%，农民人均纯收入增长6.5%。全社会固定资产投资116.4亿元，增长35.3%。新建续建“568”计划项目225个，投资62亿元；30个重点工业项目当年投资14.6亿元。华电六安电厂一期一号机组并网发电，江汽齿轮系列汽车变速器、应流集团2万吨铸钢一期工程、世林新型高效节能灯、明都线缆、恒兴烤鳗、金峰酒业、将军磁业、天裕米业等项目竣工投产，霍邱铁矿开发和金安钢铁、江淮电机、星星轻纺、海洋羽绒、建来化工、集伟鞋业等扩建项目建设成效明显。济广、宁西、合阜高速公路六安段施工加快，沪汉蓉铁路和六武高速公路六安段开工建设，新建改建农村公路895公里，金寨村村通油（水泥）路试点取得明显成效。白莲崖水库成功截流，流波电站建成运营，城西湖蓄洪大堤加固和正南淮堤、临王段、淠河、史河应急加固工程不断完善，城市防洪二期工程进入扫尾阶段，郑家湾水电站和淮河周集港开工建设，淠河综合治理全面启动，行蓄洪区移民建房加快推进。完成中心城市建设投资16.7亿元，续建、新建重点工程50项。大别山革命历史纪念园、北塔公园、淠河路、云露街等重点工程相继竣工。新建安置房42万平方米。汽车客运站场成功搬迁，拆除违法建设取得阶段性成果。各县城、重点镇基础设施建设加快推进，功能逐步完善。电力、通信等建设不断加强。
　　改革开放不断深化，经济活力明显增强。国有工业企业改制扫尾加快，长江股份股权分置改革顺利完成。市属国有流通企业改制进展明显，省“万村千乡”流通网点年度试点任务超额完成。全年新增私营企业562户、个体工商户6731户、注册资金7.1亿元。城市信用社加入徽商银行，市中小企业投资担保公司挂牌运营。大力开展多形式招商，全年到位境内资金57亿元、实际利用外资5375万美元，分别增长22.8%和22%；新批外资企业22户，增长22.2%。进出口1.86亿美元，增长57.7%。
　　经济社会统筹发展，和谐六安建设扎实推进。文明创建活动深入开展，获省创建文明城市工作先进市。科技创业服务、科技专家大院和科技特派员工作初见成效。教育“两基”成果巩固提高，贫困家庭中小学生“两免一补”政策得到落实，改造农村中小学危房20万平方米，职业教育和民办教育发展加快；各类高中教育得到加强，六安一中本科录取率、600分以上高分段人数均列全省单校第一。成功举办了市首届文化产业项目推介会，公布了首批市级重点文物保护单位。公共卫生综合防治能力得到加强，新型农村合作医疗试点成果初步显现。广播电视、群众体育、新闻出版等事业健康发展，邮政收入完成率、增长率位居全省第一。面对13号台风突然袭击，紧急转移安置灾民34.4万人，全力组织灾后重建，保证了灾区群众有饭吃、有衣穿、有房住、无疫病流行。全年新增城镇就业2.68万人，城镇登记失业率4.1%。社会保险“扩面征缴”有序开展，企业离退休人员基本养老金和失业人员失业金按时足额发放，城市“低保”、农村特困群众救助政策得到落实。劳务输出157万人，培训农村劳动力5.5万人。实现整村脱贫190个，贫困人口进一步下降。农村安全饮用水工程稳步推进，江淮分水岭综合治理、农业综合开发深入开展。环境保护不断加强，造林绿化又有新进展，砂石等资源开发成效明显。大别山（六安）国家地质公园申报成功，红色旅游扎实推进。关爱女孩平抑出生人口性别比工程深入实施，人口出生率11.9‰。“平安六安”创建活动全面开展，社会治安综合治理深入推进。整顿和规范市场经济秩序取得积极成效，安全生产形势总体稳定，事故起数、死亡人数分别下降8.1%和14.6%。信访条例得到深入贯彻，开门接访、带案下访卓有成效。 “四五”普法通过验收，国防动员、民兵预备役工作稳步推进，再次荣获“全省双拥模范城”称号。第一次全国经济普查顺利完成，第二轮修志加快启动，妇女、儿童、老龄和残疾人事业不断发展，审计、统计、档案、对台、外事侨务、民族宗教、人防、地震、气象等工作都取得了新成效。 
　　政府建设得到加强，行政能力不断提高。保持共产党员先进性教育活动扎实开展。依法行政全面推进。自觉接受人大依法监督和政协民主监督，全年办结人大议案6件、代表建议96件，政协建议案2件、提案181件。行政许可实施项目和实施主体得到清理，专家咨询、社会公示、社会听证等制度逐步健全，电子政务加快发展。“三型机关”创建活动取得阶段性成果，行政效能不断提高。市政务服务中心功能明显提升，全年受理各类事项5.3万件，按时办结率达100%。
　　各位代表！发展令人振奋，成绩来之不易。我们深切感受到：在六安这样一个发展中的地区，要实现又快又好的发展，必须始终坚持从市情出发，坚定不移地走符合六安实际的发展路子；必须始终坚持解放思想、开拓创新，用改革的思路、创新的办法破解发展中的难题；必须始终坚持突出重点，集中力量办大事，以重点突破带动整体推进；必须始终坚持以人为本，充分调动和激发全市人民加快发展的主动性、积极性和创造性。
　　我们还清醒地看到，当前发展中还存在许多困难和问题。突出表现在经济总量不大，人均水平较低；经济结构仍然不优，工业化、城镇化水平和经济外向度不高；经济自主增长能力不足，支柱产业不壮，缺乏大企业、大项目支撑；农业基础仍然脆弱，农民持续增收的机制尚未形成；财政增收压力较大，收支矛盾较为突出；部分群众生活比较困难，就业再就业形势严峻，社会保障体系有待完善；政府机关作风和效能有待进一步改善等。对此，我们一定高度重视并认真加以解决。
　　二、坚持以科学发展观为指导，推动“十一五”跨越式发展
　　“十一五”是全面建设小康六安十分关键的五年，也是六安实现奋力崛起必须大有作为的五年。按照市第二次党代会和市委二届二次全会的总体部署，“十一五”全市经济社会发展的指导思想是，高举邓小平理论和“三个代表”重要思想伟大旗帜，认真落实科学发展观，进一步更新观念、深化改革、扩大开放，推动经济体制转换、增长方式转变、产业结构转型，实施“东向发展”战略，以中心城市发展为先导，以工业化为核心，以招商引资为突破口，以“568”计划为抓手，强力推进工业化、城镇化、农业产业化和信息化，实现“三大跨越”，推进六安崛起，加快全面建设小康六安、构建和谐社会的进程。  
　　推进六安跨越式发展，必须全面落实科学发展观，牢固树立解决六安所有问题在于加快发展的观念，不分心、不走神、不争论，确保生产总值年均增长12%以上、财政收入同步增长，2010年生产总值总量及人均水平和财政收入在2005年基础上翻一番；城镇居民人均可支配收入、农民人均纯收入分别年均增长8%和6%，2010年突破11000元和3000元。为此，必须突出以下六大重点：
　　（一）积极实施东向发展战略。进一步优化区域经济发展的空间取向和布局，主动承接“长三角”、融入合肥经济圈，发挥比较优势，整合优质资源，找准产业定位，加强战略合作。全面推进观念东学、体制东接、资金东引、产业东连，在对接中壮大，在开放中发展。
　　（二）强力推进经济结构战略性调整。坚定不移地走工业强市之路，促进经济结构全面改善。突出钢铁、汽车零部件、纺织服装、农副产品深加工、电力能源、医药化工六大工程，推进产业相对集中、企业相对集群，千方百计确保五年工业项目投入超300亿元，规模工业增加值年均增长25%以上，力争培育年销售收入超10亿元企业10家以上，其中20亿元以上5家，基本形成工业主导型经济增长格局。按照区域化布局、规模化生产、科学化种养、订单化销售、市场化经营的原则，重点发展优质粮油、皖西白鹅、奶牛、名优茶、六安大麻、经济林果、优质蚕茧等主导产业，着力培育一批竞争力强的龙头企业和企业集群，新增国家级龙头企业3家、省级10家以上、市级50家以上，发展农产品营销队伍30万人，农产品优质率和加工转化率分别达到80%和50%以上，全面提升农业产业化水平。加快发展第三产业，构建大别山区域性商贸、物流、休闲娱乐中心和旅游目的地，加快建设“数字六安”应用、支撑和基础设施体系，全面推进金融服务、社区服务、中介服务等现代服务业发展。
　　（三）大力实施城镇化战略。按照点轴发展模式，以六安城区为中心，以各县城和叶集试验区为次中心，以重点镇为骨干，以方便快捷的交通为纽带，加快形成“一城率先崛起、六方竞相呼应、群星争辉显亮”的放射型网状式城镇体系，城镇化率达到38%以上。进一步加快中心城市建设，坚持老城改造与新城建设相结合，人口扩张与产业发展相适应，形成60平方公里、50万人口框架。按照规模化、生态化、宜居化要求，完善城市功能，提升管理水平，打造城市品牌，进一步发挥中心城市在区域发展中的辐射带动作用。
（四）加快建设社会主义新农村。坚持以规划为先导，围绕建设新村镇、发展新产业、培育新农民、树立新风尚、建立新机制，启动百村示范工程，推进社会主义新农村建设。进一步加大治水安民力度，完成治淮阶段性任务，积极实施淠河、史河等主要内河及洼地的综合治理，推进江淮分水岭综合治理开发。进一步做好改水、改路、改厕、改圈、改厨工作，实现村村通油（水泥）路，大力实施农村小康环保行动计划，全面改善农民生产生活条件和农村面貌。继续深化农村综合改革，积极发展农村公共事业，强化农业和农村发展支撑措施，建立农村基层工作和农村公共产品供给新机制。大力推进扶贫开发，“十一五”末绝对贫困人口减少到5万人以下。
　　（五）全面实施创新推动战略。坚持以观念创新为先导，以体制机制创新和科技创新为突破口，培育尊重创新、鼓励尝试、宽容失败的创新文化。围绕完善社会主义市场经济体制，以更大的魄力深化改革。积极培育高新技术企业，增强企业自主创新能力。创新投资方式，确保五年完成固定资产投资850亿元以上。建立完善银企合作新机制，促进企业直接融资，力争新增2-3家企业上市。实施全民创业行动，着力营造良好的舆论、信用、体制、执法、金融环境，在全市形成百姓创家业、能人创企业、干部创事业的生动局面。
　　（六）着力构建和谐六安。实施积极的就业政策，完善和落实各项扶持措施，确保五年新增城镇就业12万人、城镇登记失业率控制在4.5%以内。不断完善社会保险制度，巩固城市“低保”，逐步开展农村“低保”，推进“五保老人之家”建设，发展慈善事业，基本实现老有所养、弱有所助、病有所医、贫有所济。大力推进科教兴市和人才强市战略，逐步促进人力资源向人力资本转变。扩大公共卫生体系覆盖面，普及新型农村合作医疗，实现人人享有初级卫生保健。稳定低生育水平，人口自然增长率控制在6.9‰以内。推进文化体制改革，繁荣文化事业，发展文化产业。进一步提高保障公共安全和处置突发事件的能力，大力加强和改进信访工作，认真落实社会治安综合治理各项措施，打造“平安六安”。建立健全生态市建设的服务网络和科技支撑体系，在加快发展中保护好“青山绿水”，努力建设资源节约型和环境友好型社会。
　　各位代表! 未来五年，机遇与挑战并存，机遇大于挑战。只要我们抓住机遇，乘势而上，团结奋斗，开拓创新，就一定能够在日趋激烈的区域经济竞争中赢得主动，就一定能够在全面建设小康六安的伟大事业中创造辉煌！
　　三、开拓创新，扎实做好“十一五”开局之年的各项工作
　　今年是实施“十一五”规划的第一年，我们必须扎实做好各项工作，为“十一五”发展奠定坚实基础。主要预期目标是：生产总值增长12%；财政收入同步增长；全社会固定资产投资增长20%；社会消费品零售总额增长12%；进出口总额增长20%；城镇登记失业率控制在4.2%以内；城镇居民人均可支配收入增长8%；农民人均纯收入增长6%；人口自然增长率控制在6.5‰以内。为此，要着力抓好十个方面工作：
　　（一）加快工业化进程，大力提升工业发展水平。深入开展“发展工业学霍山”活动，以重点项目为着力点，以骨干企业为支撑点，确保规模工业增加值增长25%以上并高于全省平均水平。
　　着力抓好工业重点项目和骨干企业。项目是投入的载体、发展的基础，大项目催生大企业，大企业造就大产业。因此，要加快推进霍邱铁矿开发和深加工、华电六安电厂和50万伏输变电工程，继续抓好长江精工工业园、华源纺织二期、华源制药液体制剂、东磁集团磁性材料、大昌和诺普等铁矿采选、大别山科技公司油茶籽深加工、国能生物质发电、大海公司淀粉生产线、星星轻纺大麻棉质化、华夏汽车零部件、集伟鞋业扩建等重点项目，开工建设迎驾集团高档水晶玻璃和电器生产、应流集团碱性树脂砂铸钢件、鸿福晶体公司扩建、英明化工公司精细化工、建来集团系列产品开发、新中天公司旋窑生产线、龙津集团高吸水树脂、三农集团粮油饲料及副产品精深加工、天业集团粮食深加工、宝祥公司家禽加工等项目。引导工业骨干企业制定和完善中长期规划，把技术进步、技术改造和规模扩张作为企业发展的生命线，力争全年销售收入超亿元企业达到40家以上，其中超3亿元15家、超5亿元5家。鼓励骨干企业提高技术创新能力和实施品牌战略，健全完善骨干企业联系制度和服务“直通车”制度。
　　发展中小企业。继续实施“中小企业促进工程”，引导集群式发展。制定更加灵活和具体的政策措施，建立健全服务体系，通过配套协作、引进重组、自主创新等途径，积极发展一批成长性好、科技含量高、有市场前景的中小企业，力争新增规模企业70户以上。
　　加快开发区、试验区和工业园区建设。把扩大招商引资与提高产业集中度、加强基础设施建设与提高工业配套化水平统一起来，不断增强产业聚集能力，全面提升园区开发水平。六安开发区力争引资5亿元以上，其它各工业园区力争引资4亿元以上。进一步建立健全园区公共服务网络，加快标准化厂房建设，清理清退圈而不用、占而不动的闲置土地，提高土地利用效益。叶集试验区要发挥体制机制优势，实现更快发展。
　　（二）大力发展农村经济，全面启动社会主义新农村建设。以发展农村经济为中心任务，促进农民持续增收，协调推进农村经济、政治、文化和社会建设。
　　认真落实各项惠农政策。确保中央和省出台的各项惠农政策全面落实到位。不断增加对农业和农村的投入，各级财政支农资金增量要高于上年。加强对农民的经营性、服务性收费监管，强化农资市场管理，巩固农村税费改革成果，支持中直六安粮库等国有企业落实好国家粮食托市收购政策。
　　提升农村经济发展水平。大力推进农业产业化，重点扶持三农集团、庆发集团、天业集团、寿县雨润、华安达集团、海洋羽绒、华园乳业等龙头企业，积极推进与市外大型龙头企业的合资合作。巩固现有并组建新的市级协会，加快发展农民专业合作组织。大力发展现代农业，推广良种良法，加快建设西海农业生态园等一批示范工程。突出发展白鹅、奶牛、生猪、肉鸡等养殖小区，建设库区湖区优势水产品养殖基地和郊区蔬菜基地。完善农产品质量检测认证等社会化服务体系，抓好农业标准化建设。大力发展林业产业，推进林业资源深加工。进一步强化培训、认真引导、科学组织，继续大力发展劳务经济、打工经济。
　　加强农村基础设施建设。加大淮河治理力度，推进淠河等内河综合治理，加强机电泵站建设，提高防洪排涝标准。全面推进村村通油（水泥）路工程，启动1000公里以上农村公路改造，完成15项以上渡改桥工程。加快农村饮水安全、电网改造、生态家园等工程建设，实施乡村重点绿化工程，深入推进江淮分水岭综合治理开发和土地开发整理。积极争取并落实省“千村百镇”试点，实施村庄整治。不断完善农业综合开发新机制，充分发挥项目区的平台作用。进一步用好用活“一事一议”制度，鼓励和引导农民搞好农村公益事业建设，特别要引导农民对直接受益的基础设施投工投劳。
　　发展农村公共事业。坚持以培训农民、培育大户、培养农民经纪人队伍为重点，逐步造就有文化、懂技术、会经营的新型农民。加强农村薄弱学校建设，进一步落实“两免一补”政策。推进广播电视村村通工程，加快发展农村文化事业。加强农村公共卫生和基本医疗服务体系建设，扩大新型农村合作医疗制度试点，高度重视农村大病救助工作。抓好高致病性禽流感等重大动物疫病和人畜共患病防控，推进基层畜牧兽医体制改革。加大扶贫开发力度，实现整村脱贫170个。完善农村特困群众救助制度，进一步做好农村“五保户”和优抚对象的生活保障工作。
　　（三）继续推进中心城市建设，不断改善城乡基础设施状况。突出中心城市的龙头地位，协调推进城镇建设，确保城镇化率提高2个百分点。
　　加大中心城市建设和管理力度。广泛吸纳社会资本参与城市建设和管理，使城市建设市场化、城市资源资本化、城市经营公司化、投资主体多元化、政府调控规范化。继续抓好皖西大道东段、大别山西路、长安北路、佛子岭西路大桥、污水管网二三期等重点续建项目。加快建设市老年活动中心、中心步行街及医疗废物集中处置中心，开工建设市博物馆、全民健身活动中心。综合整治淠河总干六安城区段和城区防洪排水体系。推进旧城改造，建成20万平方米安置房。加强社区建设，提升服务功能。进一步完善城市管理体制，提高管理效能。加大拆除违法建设力度，建立长效管理机制。强化市容市貌整治，大力整顿交通秩序，深入实施绿化、净化、亮化、美化工程，提高城市品位。各县城和中心镇也要运用市场化的办法加快建设步伐，提升城镇开发水平，扩大规模，完善功能，改善环境，提高品位。坚持产业兴镇，提高城镇集聚和承载能力，发展各具特色的城镇经济。
　　抓好重点基础设施建设。全力支持和积极参与沪汉蓉铁路及济广、宁西、合阜、六武高速公路六安段建设，加大高速公路连接线项目的争取和建设力度，开工建设经三路新淠河大桥和宁西高速公路城北连接线。加快霍众路工程进度，改造霍寿路，建设瓦（埠）高（刘）路、李（台）石（集）路等重要县道。加大治淮工程建设力度，推进佛子岭抽水蓄能电站和淮河周集港建设，加快白莲崖水库、淠史杭续建配套、内河治理、城区防洪和行蓄洪区安全建设及移民迁建等工程进度。
　　（四）大力发展第三产业，提高服务业水平和层次。坚持以市场化、社会化、产业化为目标，提升传统服务业，加快发展现代服务业，着力构建区域性商贸物流休闲中心。
加快发展旅游业。坚持统筹协调，突出资源整合，积极发展红色旅游、生态旅游和古文化旅游，大力推进大别山（六安）国家地质公园建设，着力打造大别山精品旅游区。加快实施旅游重点项目，支持天堂寨、万佛湖申报4A级景区，全面启动“139”红色旅游精品线路建设。改善旅游交通等基础设施，加强对旅游从业人员培训和管理，增强服务能力。
　　提升商贸流通业。加快城市商业网点规划编制工作，推进综合和专业市场、物流基地、大型商场和高星级宾馆建设，丰富休闲服务业，提升传统服务业，大力发展连锁经营、物流配送和电子商务等新型业态，加快国际汽车城、新都会大润发超市、西商食品工业园、火车站南物流集散地等项目建设。继续做好省“万村千乡”试点工作，建成一批配送中心和乡村连锁店，逐步形成高效畅通的农村商品流通网络。推进房地产二、三级市场发展，逐步健全住房公积金制度。规范完善会计、评估等中介服务组织，积极发展代理、典当、拍卖等服务业。大力开拓出口市场，扶持出口大户，落实和用好退税政策。
　　发展文化产业。深度开发特色文化资源，培育一批重点文化企业，注重用高新技术开发文化产品。深化文化体制改革，积极推进文化团体与企业联姻，大力开展项目招商，放宽项目准入条件，鼓励和引导民间投资兴办文化产业。
　　做好财税工作。进一步深化财政体制改革，完善国库集中支付制度。坚持开源节流，严格税收征管，注重科学征管。加强政府采购和国有资产管理。完善财政分配制度，推进县乡财政振兴工程。加强财政资金使用的监督管理，促进机关事业单位的公平分配。
　　（五）加大招商引资力度，全面改善发展环境。紧紧抓住发达地区产业转移的机遇，加大招商引资力度，增强工作实效性。
　　提升引资质量和水平。坚持东向发展，主攻长三角，紧盯珠三角，关注环渤海、港澳台和日韩等地区，大力实施产业招商，注重开展资源招商。认真筹备参加徽商大会、中国投资贸易洽谈会等重大招商活动。充分调动企业对外合资合作积极性，积极开展以商招商、部门联合招商和小分队招商，组建各级专业招商队伍，开展驻点招商。完善招商政策体系和评价体系，强化“一把手”负责制和目标考核责任制，严格兑现奖惩。
　　大力加强投资环境建设。坚持“人人都是投资环境，处处都是六安形象”，努力营造“进了六安门，就是六安人；到了六安市，处处好办事”的社会氛围。建立健全首问负责、服务承诺、全程办理、责任追究等制度，深入开展“发展环境万人评”活动。进一步深化行政审批制度改革，切实加强政务服务中心建设，提高办事效率和水平。推进信用六安建设，努力营造诚实守信的信用环境。整顿和规范市场经济秩序，维护投资者合法权益。加大对侵害投资者权益的案件查处力度，强化领导责任制和责任追究制。
　　（六）积极开展全民创业，加快发展县域经济。把全民创业作为奋力崛起、振兴县域经济的重要抓手，广泛发动、深入组织，把领导的思路变成群众的共识，把领导的决心变成群众的行动，让每个人的思想都活跃起来，让每个层面的激情都焕发出来，汇成发展的强大动力和活力。全面落实国家和省有关鼓励支持城乡居民自主创业、发展民营经济的各项政策措施，建立健全以信息、技术、市场、资金、人才和政策服务为主要内容的创业平台。全面推进“双向创业”，大力实施“凤还巢”工程，积极发展“草根经济”。引导金融机构加大支持力度，开展各种形式的小额信贷，进一步提高对民营企业的贷款比重。坚持区别对待、分类指导，引导县区立足比较优势，以工业化为核心，以招商引资为突破口，着力培育特色经济、主攻民营经济、壮大园区经济、发展旅游经济。鼓励和支持发展基础较好的，乘势而上，努力跻身全省经济强县；发展水平中等的，增强进取意识，瞄准强县，奋起直追；发展暂时滞后的，振奋精神，激发活力，尽快缩小差距。
　　（七）全面推进改革创新，增强经济发展内在动力。坚持以建立健全社会主义市场经济体制为目标，逐步解决影响发展的体制性障碍和深层次矛盾，为经济社会发展提供强大动力。 
　　全面推进各项改革。深化农村综合改革，逐步建立以服务“三农”为核心的农村工作新机制。加快市直国有工业企业改革扫尾，全面完成县区国有工业企业改革任务，加大国有商贸流通企业改革力度，推动建设和交通运输系统企业改革，探索农业、林业和水利等系统企事业改革。进一步清除非公有制经济发展的体制性障碍，放宽市场准入，保障非公有制经济合法权益。
　　提高融资能力。加快培育多元化投资主体，全力支持企业直接融资。重视和支持金融工作，努力改善金融生态，建立金融协调机制，完善并落实鼓励金融机构支持地方经济发展的政策措施。推动有条件的农村信用社组建农村合作银行。各金融机构要强化服务意识，树立“发展地方经济我有责，我与地方发展共兴衰”的理念，在发展中防范风险，在扩张信贷规模中赢得效益，在解决中小企业融资难等问题上有所作为。充分发挥市城投公司、交基公司等融资平台作用。支持市中小企业投资担保公司发展，设立市中小企业发展创新基金。
　　积极创新项目工作机制。充分利用各种条件，挖掘各方潜力，动员一切资源，努力形成竞相抓项目、促项目、上项目的浓厚氛围。创新项目生成机制，全面加强同院校、科研院所的联系，力争新增项目储备规模200亿元以上。加强项目培训工作，培育项目工作队伍。加大财政对项目工作支持力度，鼓励社会投资开展项目前期工作。落实重大项目领导联系制和全程服务制度，及时解决项目建设中的突出问题。
　　（八）加强统筹协调，全面推进政治建设、文化建设和社会建设。加强民主法制和精神文明建设，把社会事业发展摆到更加突出的位置，不断提高人民群众思想道德素质、科学文化素质。 
　　加强民主法制建设。坚持依法治市，大力推进“五五”普法。推进依法行政，抓好综合执法试点，开展行政执法依据清理，逐步健全在行政诉讼中机关法定代表人出庭应诉制度。强化行政执法监督，严格落实行政执法责任制、评议考核制和执法过错追究制。重视人口老龄化问题，实施妇女、儿童发展规划，维护残疾人合法权益。巩固和扩大基层民主，支持工会、共青团、妇联、科协等群众团体工作，充分发挥职代会、村（居）委会作用。
　　全面推进精神文明建设。深入开展文明城市、文明村镇、文明单位和文明家庭创建活动，制定并实施文明市民守则，增强全社会“爱我六安、爱我家园”意识，争创全省文明城市。抓好青少年思想品德和心理素质教育，整治网吧、图书、音像市场和文化娱乐场所，营造有利于青少年健康成长的思想文化环境。加强科普宣传，加大殡改力度，提高全社会文明程度。  
　　深入实施人才强市和科教兴市战略。牢固树立“人才是第一资源”观念，加大人才培养和引进力度，重点加强经营管理人才、专业技术人才和实用人才队伍建设，建立有利于优秀人才脱颖而出的选拔任用机制。市科技创业服务中心、专家大院和科技特派员创业行动要求实效、出成果。巩固提高“两基”成果，加大政府对义务教育的投入力度，大力发展学前教育和高中教育，支持办好高等教育。加强中小学校布局调整和寄宿制学校建设，改造中小学D类危房10万平方米以上。严格规范教育收费，完善家庭困难学生资助制度。
加快生态市建设。继续抓好淮河、巢湖流域污染治理和城镇环境综合治理，全面实施生态市建设规划，争创省级环境保护模范城市。大力推进省“百镇千村万户生态示范工程”，积极做好生态建设综合示范基地申报工作，提高森林覆盖率，实行资源有限、有序和有偿开发，加强生产要素的节约和综合利用，加快发展循环经济。
　　发展各项社会事业。安排社会事业发展贴息资金，加快社会事业项目建设。落实人口和计划生育奖励政策，稳定低生育水平，着力解决出生人口性别比偏高问题。推进文化基础设施建设，举办中国六安（大别山）首届红军文化节。完善公共卫生和医疗服务体系，提高疾病预防控制和医疗救治服务能力，做好艾滋病、结核病和乙肝等重大传染性疾病防治工作。积极备战省十一届运动会，深入开展全民健身运动。推进第二轮修志和历代旧志整理工作，加强统计基层基础建设，改革档案管理模式。认真开展全民国防教育，加强国防后备力量和人防建设，争创全国双拥模范城。
　　（九）高度重视民生，着力构建和谐社会。把群众利益放在首位，高度重视并及时解决群众特别是困难群众生产生活中的实际问题。 
　　推进就业再就业工作。把扩大就业作为经济社会发展的优先目标，确保新增城镇就业2.35万人，其中下岗失业人员再就业1.2万人，重点帮扶“4050”困难群体再就业。加强街道社区劳动保障平台建设，建立就业创业基地，启动创业帮扶工程，积极推广组织起来就业。认真落实再就业优惠政策，逐步将集体企业下岗失业人员纳入再就业扶持范围，做好进城求职农民和被征地农民就业服务工作。加强失业调控，统筹城乡劳动力就业。
　　加快社会保障体系建设。完善社会保险政策，扩大覆盖面，加强基金征管，增强保障能力。改革养老金计发办法，逐步做实个人账户，实行养老保险市级统筹。做好事改企单位和机关事业单位临时工作人员养老保险工作，推进企业退休人员社会化管理。强化城市“低保”动态机制，探索建立被征地农民生活保障制度。
　　切实维护社会稳定。加强信访工作，坚持和完善各级领导信访接访日制度，依法处理各类社会矛盾。进一步加强法律援助，保障弱势群体合法权益。坚持“人民至上、生命至上、安全至上”的原则，抓好安全生产基础工作，加强食品药品、环境等安全监管，严格落实责任制和责任追究制。加强水文、气象、地质、地震等灾害预测预报工作，提高防灾减灾救灾能力。建立健全应急机制，不断提高应对突发事件的能力。加快社会治安防控体系建设，强化社会治安综合治理，努力形成社会安定、环境安全、百姓安宁的和谐局面。 
　　（十）全面加强政府建设，提高行政效能。各级政府及其各个部门必须坚持科学行政、民主行政、依法行政，全面加强效能建设，切实提高施政水平。进一步解放思想、更新观念，善于用改革的思路、创新的办法、市场的手段来解决矛盾、处理问题、促进发展。加快职能转变，继续减少和规范行政审批。发展电子政务，提高政府工作透明度。努力创建“三型机关”，加强作风建设，确立“部门就是服务、服务就是环境”的观念，强化为基层、为企业、为老百姓服务的意识，把企业发展的难点作为机关服务的重点，把老百姓关注的热点作为改进工作的着力点，切实做到不让一个商机在我这里丧失、不让一个工程在我这里延误、不让一件有利发展的事情在我这里卡壳、不让一个投资者在我这里受冷落。要精简会议和文件，多干实事，少讲空话，不提或少提新口号，重在抓落实。抓落实要下劲、要具体、要有形、要量化，重结果，看变化。加强廉政建设，强化审计监督，进一步规范政府采购、土地出让、工程招标、产权交易等行为，逐步健全教育、制度、监督并重的惩治和预防腐败体系。各级政府要自觉接受党的领导和人大的法律及工作监督、政协的民主监督，坚决执行党委决定，认真办理人大议案、代表建议和政协建议案、委员提案。全面贯彻《公务员法》，加强公务员队伍建设，教育和引导广大公务员树立正确的理想信念和高尚的道德情操，自觉做到为民、务实、清廉，始终保持奋发有为的蓬勃朝气、开拓创新的昂扬锐气、清正廉洁的浩然正气，努力开创政府工作新局面。
　　各位代表！站在六安“十一五”发展新的历史起点上，我们深知责任重大，倍感使命光荣。全市人民对政府的信任和支持，给了我们无穷的力量和必胜的信念。让我们在中共六安市委的坚强领导下，坚定信心，与时俱进，开拓进取，奋发有为，为圆满完成今年和“十一五”规划的各项目标任务、实现“三大跨越”、加快六安崛起而努力奋斗！
